# Cutting off the fuel supply to calcium pumps in pancreatic cancer cells: role of pyruvate kinase-M2 (PKM2)

**DOI:** 10.1038/s41416-019-0675-3

**Published:** 2019-12-10

**Authors:** Andrew D. James, Daniel A. Richardson, In-Whan Oh, Pishyaporn Sritangos, Thomas Attard, Lisa Barrett, Jason I. E. Bruce

**Affiliations:** 1 Division of Cancer Sciences, Faculty of Biology, Medicine & Health Sciences, The University of Manchester, Michael Smith Building, Manchester, M13 9PT UK; 2 Present Address: Division of Cancer Sciences, Department of Biology, University of York, Heslington, York YO10 5DD UK

**Keywords:** Cancer metabolism, Calcium signalling

## Abstract

**Background:**

Pancreatic ductal adenocarcinoma (PDAC) has poor survival and treatment options. PDAC cells shift their metabolism towards glycolysis, which fuels the plasma membrane calcium pump (PMCA), thereby preventing Ca^2+^-dependent cell death. The ATP-generating pyruvate kinase-M2 (PKM2) is oncogenic and overexpressed in PDAC. This study investigated the PKM2-derived ATP supply to the PMCA as a potential therapeutic locus.

**Methods:**

PDAC cell growth, migration and death were assessed by using sulforhodamine-B/tetrazolium-based assays, gap closure assay and poly-ADP ribose polymerase (PARP1) cleavage, respectively. Cellular ATP and metabolism were assessed using luciferase/fluorescent-based assays and the Seahorse XFe96 analyzer, respectively. Cell surface biotinylation identified membrane-associated proteins. Fura-2 imaging was used to assess cytosolic Ca^2+^ overload and in situ Ca^2+^ clearance. PKM2 knockdown was achieved using siRNA.

**Results:**

The PKM2 inhibitor (shikonin) reduced PDAC cell proliferation, cell migration and induced cell death. This was due to inhibition of glycolysis, ATP depletion, inhibition of PMCA and cytotoxic Ca^2+^ overload. PKM2 associates with plasma membrane proteins providing a privileged ATP supply to the PMCA. PKM2 knockdown reduced PMCA activity and reduced the sensitivity of shikonin-induced cell death.

**Conclusions:**

Cutting off the PKM2-derived ATP supply to the PMCA represents a novel therapeutic strategy for the treatment of PDAC.

## Background

Pancreatic ductal adenocarcinoma (PDAC) has one of the poorest survival rates (5-year survival: 3–6%) and is predicted to be one of the leading causes of cancer-related deaths.^1,2^ PDAC typically progresses in the absence of clinical symptoms,^3,4^ and upon diagnosis, the tumour has often metastasised.^5^ Moreover, PDAC is largely resistant to chemotherapy, due in part to the dense desmoplasia that restricts drug delivery to the tumour.^6^ Even with early detection and improved drug delivery, the design of novel drugs that selectively kill PDAC cells must remain a central research strategy.

PDAC cells undergo a switch from mitochondrial to glycolytic metabolism (Warburg effect^7,8^), which facilitates numerous cancer hallmarks, including cell proliferation, invasion and resistance to apoptosis.^9^ Our previous studies show that this increased glycolytic rate is also important for fuelling the ATP-dependent plasma membrane calcium pump (PMCA), as inhibition of glycolytic ATP production in PDAC cells causes cytotoxic Ca^2+^ overload and cell death.^10,11^ This dependency of the PMCA on glycolytic ATP in PDAC represents a potential therapeutic avenue, and understanding the underlying molecular mechanisms may reveal novel therapeutic targets against which novel drugs can be designed.

Pyruvate kinase-M2 (PKM2) is a major oncogenic ATP-generating glycolytic enzyme and is particularly overexpressed in pancreatic cancer.^12–17^ PKM2 has a low catalytic activity, producing a bottleneck at the terminal end of glycolysis.^12,18^ This is advantageous to cancer cells as it results in the buildup of glycolytic intermediates, which are then utilised in nucleotide and lipid biosynthesis to aid rapid proliferation. However, this also presents a potential weakness of the cancer cell, as PKM2 may increase biosynthesis at the expense of ATP production.^12,18^ This is particularly important in the context of ATP-dependent pumps at the plasma membrane. In erythrocytes and smooth muscle cells, glycolytic enzymes localise at the plasma membrane in close proximity to the PMCA.^19,20^ These enzymes support an ATP-rich microdomain around the PMCA, thus providing an efficient ATP supply with which to preserve its function. If extrapolated to cancer cells, the association of at least a proportion of total PKM2 with the plasma membrane could provide a privileged ATP supply to the PMCA; this might be particularly important in circumstances where global cytosolic ATP supply is limited, such as those where the low catalytic activity of PKM2 favours biosynthesis over glycolytic ATP production.

Shikonin is one of the most potent and selective inhibitors of PKM2, with a 10–20-fold higher selectivity for PKM2 vs PKM1 and PKL,^21^ and is reported to exhibit antimicrobial, anti-inflammatory as well as numerous anticancer effects.^21–25^ Although shikonin also exhibits numerous PKM2-independent effects, these effects generally occur at much higher concentrations (supramicromolar) over prolonged treatment periods (>24 h).^26^ However, most of the reported acute effects (up to 1 h) of lower concentrations of shikonin (sub-micromolar) are due to specific inhibition of PKM2. This makes shikonin a useful tool to study the PKM2-mediated ATP supply to the PMCA in PDAC cells. Therefore, the current study investigated the functional coupling between PKM2 and PMCA and whether this contributes to cancer hallmark responses using a combination of acute treatment with shikonin and siRNA-mediated knockdown of PKM2. The results from data mining and survival analysis show that high PKM2 expression contributes to poor survival in patients with PDAC. Moreover, PKM2, using shikonin inhibited PDAC cell proliferation, migration and induced cell death. These effects were due in part to inhibition of glycolysis, ATP depletion, inhibition of PMCA activity and the resultant cytotoxic Ca^2+^ overload. The effects of shikonin were due at least in part to specific inhibition of PKM2; knockdown of PKM2 with siRNA also inhibited PMCA activity and prevented shikonin-induced cell death. These data suggest a functional coupling between PKM2 and the PMCA that is critical for cell survival, and implicate the PKM2-derived ATP supply to the PMCA as an important and novel therapeutic locus in PDAC.

## Methods

### Cell culture

Human PDAC cell line Mia PaCa-2 cells were cultured in humidified air (5% CO_2_ and 95% O_2_) at 37 °C in either high (25 mM, D6429, Sigma) or low (5 mM, D6046, Sigma) glucose-containing Dulbecco’s modified Eagle’s medium (DMEM). The media were supplemented with 10% FCS, 100 units/ml penicillin and 100 g/ml streptomycin.

### Data mining and survival analysis

Oncomine software (Thermo Fisher Scientific, Ann Arbor, MI) was used to generate heatmaps and access gene chip microarray expression data from the Badea Pancreas study.^25^ Kaplan–Meier survival curves were generated from the same data using PROGgeneV2 (Indiana University Purdue University, Indianapolis, IN).

### Sulforhodamine-B (SRB) assay

Cell proliferation rate was measured using the sulforhodamine B (SRB) protein stain assay. MIA PaCa-2 cells (5000 cells per well) were seeded in clear 96-well culture plates and fixed at 2–96 h using 10% (wt/vol) trichloroacetic acid (TCA) for 1 h at 4 °C. The plates were then washed with H_2_O, dried and stained using 0.057% SRB for 30 min at room temperature, rinsed with 1% (vol/vol) acetic acid and protein-bound dye solubilised with 10 mM Tris base solution (pH 10.5). Absorbance was measured at 540 nm using a BioTek^®^ Synergy HT plate reader.

### Tetrazolium-based Cell Counting kit (CCK-8)

MIA PaCa-2 cells were prepared similar to the SRB assay and then incubated for 1 h at 37 °C, 5% CO_2_ with the tetrazolium-related substrate, 2-(2-methoxy-4-nitrophenyl)-3-(4-nitrophenyl)-5-(2,4-disulfophenyl)-2H-tetrazolium (WST-8; Dojindo) yielding a coloured product whose absorbance is measured at 450 nm.

### Fura-2 fluorescence Ca^2+^ imaging

MIA PaCa-2 cells were loaded with 5 μm of fura-2-AM for 40 min at room temperature and imaged using a Nikon TE2000S microscope with ×40 oil immersion SFluor objective lens, CoolSNAP HQ CCD camera (Photometrics, Tucson, AZ) and Cairn monochromator (Cairn Research, Kent, UK), controlled by MetaFluor imaging software (Molecular Devices, Downington, PA). Background-subtracted 340- and 380-nm fluorescence images were captured with 50-ms exposure and 5 × 5 binning every 5 s, and emitted light was separated using a 400-nm dichroic with 505LP filter. The fura-2 fluorescence was calibrated into “estimated” [Ca^2+^]_i_ as previously described.^10,11^

### In situ [Ca^2+^]_i_ clearance assay

Fura-2-loaded Mia PaCa-2 cells were first perfused with Ca^2+^ + -free (0 Ca^2+^) HEPES-buffered physiological saline (HPSS) containing 30 μM cyclopiazonic acid (CPA) and 1 mM EGTA to deplete ER Ca^2+^ and activate store-operated Ca^2+^ entry (SOCE). Therefore, the addition of 20 mM Ca^2+^ caused a rapid increase in [Ca^2+^]_i_ due to SOCE, which reached a short-lived steady state, and the subsequent removal of external Ca^2+^ (HPSS containing 1 mM EGTA) caused a rapid decrease in [Ca^2+^]_i_ due to PMCA-mediated Ca^2+^ clearance.

### Luciferase-based ATP assay

MIA PaCa-2 cells (5000 cells per well) were seeded in white-walled, clear-bottom 96-well cell culture plates, and ATP content of the cells was measured using a ViaLight™ plus kit (Lonza) and a BioTek^®^ Synergy HT plate reader. Luminescence was normalised to the corresponding Sulforhodamine-B assay (SRB) values before normalising to a vehicle control.

### FRET-based cellular ATP assay (GO-ATeam)

MIA PaCa-2 cells were stably transfected with DNA encoding GO-ATeam, using GeneCellin (BioCellChallenge) followed by selection using G418 as previously described.^11^ GO-ATeam is a recombinant FRET-based (*G*reen/*O*range) fluorescent *AT*P reporter.^27^ GO-ATeam-transfected cells were imaged using the above imaging, except that cells were excited at 470 nm and emitted light at 510 and 560 nm were simultaneously collected using an OptoSplit image splitter (Cairn).^11^

### Seahorse extracellular flux (XF) analysis

Extracellular acidification rate (ECAR) was used to indirectly calculate the glycolytic rate of cells using a Seahorse XFe96 analyzer (Agilent). Cells were seeded in Seahorse 96-well microplates (Agilent) (5000 cells/well) in half the final volume of culture medium at the respective glucose concentrations. Data were processed using Wave software (Agilent) and normalised to the SRB assay. Due to the red colour of shikonin affecting the solid-state sensors, measurement of OCR was not possible.

### Cell surface biotinylation assay

This was achieved using a Pierce-Thermo Cell Surface Protein Isolation Kit Pierce (#89881) in which cell surface proteins were labelled with a cleavable biotin (Sulfo-NHS-SS-Biotin) in intact live cells for 1 h at 4 °C. Biotin-labelled protein was extracted using NeutrAvidin conjugated agarose beads, eluted using dithiothreitol and denatured at 95 °C for 5 min in SDS sample buffer, prior to separating using SDS-PAGE and western blotting for glycolytic enzymes and PMCA. Dithiothreitol precluded protein quantification from biotinylated fractions. Primary antibodies used include non-specific PMCA (PMCA NS (5F10)), PMCA1 and PMCA4 (JA9) (Thermo Pierce) and the Glycolysis Antibody Sampler Kit (Cell Signaling Technology, Kit 8337).

### Cell migration assay (Gap closure assay)

A ‘2-chamber’ Ibidi insert was pre-fitted into 12-well culture-treated plates and MIA PaCa-2 cells were seeded at 50,000 cells/chamber, and allowed to grow to 95–100% confluence for 24 h. The insert was then removed and migration of cells into the gap was monitored. Images were acquired using an Olympus IX83 inverted microscope with a 4 × /0.13 LUC PlanFL N objective lens, Orca ER Camera (Hamamatsu) and CellSens software (Olympus) and images processed using the Fiji ImageJ programme.

### PKM2 siRNA knockdown

PKM2 expression was knocked down using 50 nM ON-TARGETplus SMARTpool anti-PKM2 siRNA targeting four different PKM2 sequences (see Supplementary Methods) with non-targeting (scrambled) siRNA used as a control (Dharmacon, Colorado, USA). Transfection conditions that yielded ≥70% knockdown between 48 and 96 h, determined by qPCR with minimal toxicity, were used in the study.

### Western blot

Cells were lysed using ice-cold lysis buffer (see Supplementary Methods) and centrifuged (17,000 × *g* for 25 min at 4 °C), and supernatant protein denatured in SDS-laemmli buffer for 5 min at 95 °C. Proteins were separated by SDS-polyacrylamide gel electrophoresis (SDS-PAGE), transferred onto PVDF membranes and western blotted using the following primary antibodies: PKM2-specific rabbit monoclonal antibody (1:1000; Catalogue #13266, Cell Signalling), PKM1-specific rabbit monoclonal antibody (1:1000; Catalogue #7067, Cell Signalling), pan-PKM1/2 rabbit monoclonal antibody (1:1000; Catalogue #3190S, Cell Signalling), PARP1 rabbit antibody (1:1000; Cell Signalling, #9532) and monoclonal anti-β-actin-peroxidase antibody (1:50,000; Catalogue #A-3854-200UL, Sigma). Secondary antibodies include an anti-rabbit horseradish peroxidase-linked antibody (1:2000; Catalogue #7074S, Cell Signalling).

### Statistical analysis

All statistical analysis was conducted using GraphPad Prism (version 7) with all appropriate parametric, non-parametric and post hoc tests to determine significance indicated in each figure legend.

## Results

### PKM2 expression in PDAC correlates with poor patient survival

**Fig. 1:**
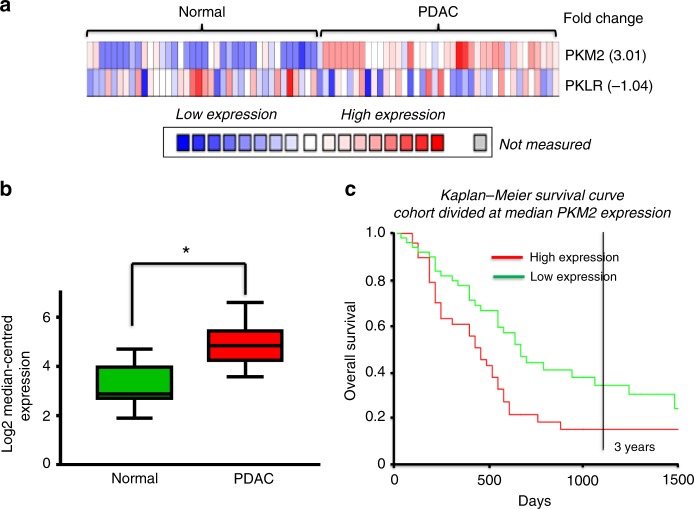
PKM2 overexpression correlates with poor prognosis in PDAC. **a** Heatmap analysis of overexpression of PKM2 vs PKLR in pancreatic tumour tissue vs healthy tissue from the same resected margin from PDAC patients. Data mining was performed on publicly available gene chip microarray data from Badea Pancreas(25) using Oncomine. **b** Box and whisker plot displaying PKM2 expression in tumour tissue vs healthy pancreas (unpaired *t* test; *n* = 39). **c** Kaplan–Meier survival analysis of PDAC patients with median-centred high vs low expression of PKM2 (hazard ratio = 1.88 (1.19–2.97), *n* = 51, *p* < 0.008; data generated using PROGgeneV2, proportional hazards analysis).

To determine whether increased PKM2 expression in PDAC tumour (vs the healthy tumour margin of the resected tissue) correlated with poor patient survival, we performed data mining of publicly available gene chip microarray data^25^ using Oncomine software (www.oncomine.com, July 2018, Thermo Fisher Scientific, Ann Arbor, MI). These data revealed that oncogenic PKM2 was overexpressed (3.01-fold, Fig. 1a; *n* = 39, *p* < 0.0001) in PDAC tissue (compared with healthy tissue), whereas expression of the non-oncogenic isoform, PKLR, was unchanged (−1.04-fold, Fig. 1a). Moreover, when the cohort was bifurcated at the median PKM2 expression, patients with low PKM2 expression lived significantly longer than patients with high PKM2 expression (hazard ratio = 1.88 (1.19–2.97), *p* < 0.008; Fig. 1c).

### PKM2 inhibitor shikonin reduces PDAC cell proliferation

**Fig. 2:**
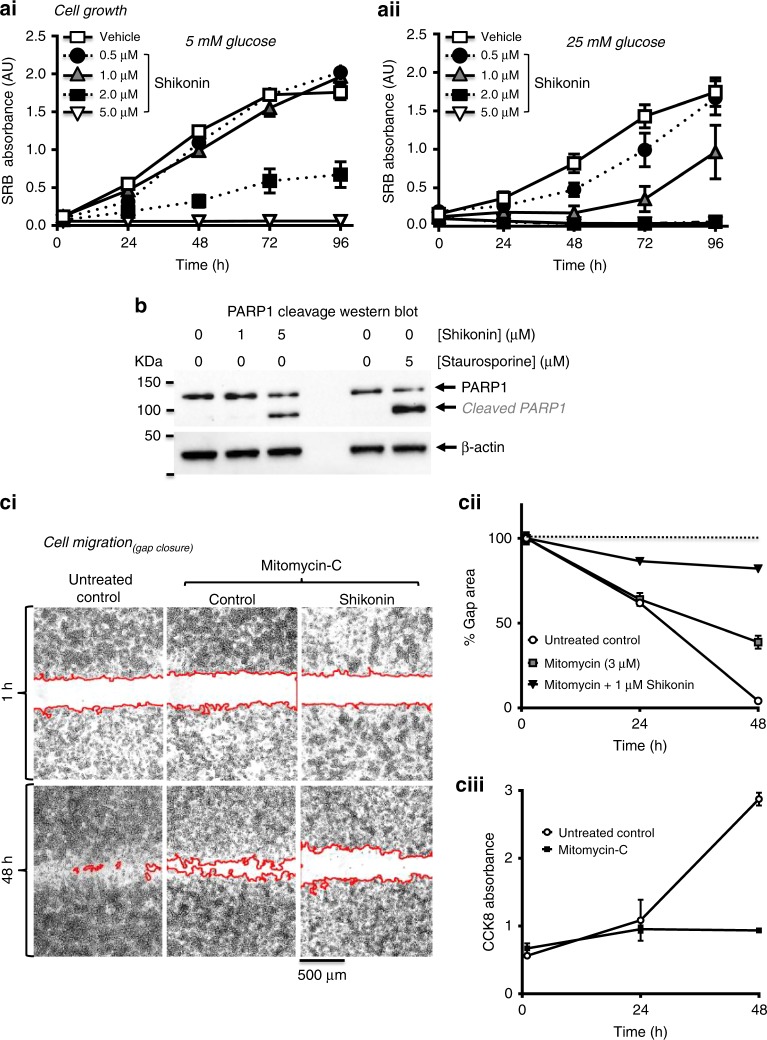
The PKM2 inhibitor, shikonin, inhibits PDAC cell growth, induces PDAC cell death and inhibits cell migration. Mia PaCa-2 cells were cultured either in 5 (**ai**) or 25 mM (**aii**) glucose-containing DMEM and treated with different concentrations of PKM2 inhibitor shikonin up to 96 h. Cell growth was measured using a sulforhodamine-B assay (SRB absorbance unit (AU)) at 2 h and every 24 h. Data were averaged across multiple repeats (8–16 per experiment) for 3–6 experiments (±SEM). **b** Western blot of Mia PaCa-2 cell lysates using anti-PARP1 antibody following treatment with shikonin (1 and 5 µM) or staurosporine (5 µM) for 6 h. Cleaved PARP1 is used as a measure of cell death and is detected as an increase in the intensity of the lower molecular weight band and a decrease in the intensity of the higher molecular weight band of full-length PARP1. β-actin is used as a loading control. **ci** Representative images of the same gap at 1 and 48 h following the removal of the Ibidi insert in the absence (untreated control) and presence of mitomycin-C (to inhibit cell proliferation) and shikonin (to inhibit PKM2). **cii** Average % gap area indicates that 1 μM shikonin significantly inhibited gap closure compared with mitomycin-C alone or untreated control cells. **cii**i CCK-8 assay confirming that 3 µM mitomycin-C inhibits cell proliferation without affecting cell viability.

To investigate the importance of PKM2 on cancer hallmark responses, we first tested the effect of PKM2 modulators on Mia PaCa-2 cell growth/viability using a sulforhodamine B (SRB) colorimetric assay and tetrazolium-based cell counting kit (CCK-8) assay. Mia PaCa-2 cells were chosen as in our previous studies, these cells were the fastest growing and exhibited the most glycolytic phenotype.^10,11^ Shikonin caused a concentration-dependant reduction in the rate of increase in SRB absorbance (Fig. 2a), indicating a significant inhibition of proliferation. The responses to shikonin were reduced when cells were cultured in restricted glucose (5 mM, Fig. 2ai) compared with standard high-glucose (25 mM, Fig. 2aii) DMEM, suggesting that shikonin is more effective at inhibiting proliferation in highly glycolytic cancer cells. The PKM2 activator TEPP-46, which promotes tetramerisation and increased catalytic activity of PKM2, had no effect on cell proliferation either in low or high glucose over 96 h (Fig. S1A, S1B).

### Shikonin reduces cell viability and induces cell death/apoptosis

As an alternative method for assessing cell growth, we used the tetrazolium-based cell counting kit (CCK-8), which provides a measure of metabolically active (viable) growing cells. The effect of shikonin (5 µM) was compared with the mitochondrial inhibitors, oligomycin (OM, 5 µM) and CCCP (4 µM), and the glycolytic inhibitor, 2-bromopyruvate (BrPyr, 500 µM). As expected, OM and CCCP had no effect, whereas BrPyr and shikonin not only inhibited cell growth but also reduced absorbance below the baseline, suggesting a reduction of viable cells or cell death (Fig. S2A). This is further exemplified when the absorbance was normalised to the corresponding untreated controls (Fig. S2B). To further investigate whether shikonin (5 µM) induced cell death, we assessed PARP1 cleavage by western blot (Fig. 2b). PARP1 is cleaved by caspase-3 and is thus a downstream effector of apoptosis.^28^ Shikonin (5 µM) induced significant PARP1 cleavage after 6 h, similar to the classical apoptosis-inducing agent, staurosporine (5 μM; Fig. 2b).

### Shikonin reduces PDAC cell migration

We next tested the effect of shikonin on Mia PaCa-2 cell migration using a gap closure assay. In untreated control cells, the gap completely closed after 48 h (Fig. 2ci and 2cii). However, to remove any confounding effect of cell growth/proliferation, cells were treated with mitomycin-C, a classical inhibitor of proliferation.^29^ Treatment of Mia PaCa-2 cells with 3 µM mitomycin inhibited growth without reducing cell viability (Fig. 2ciii) and partially reduced gap closure (Fig. 2cii), suggesting that the residual gap closure was due entirely to cell migration. Under these conditions, treatment with 1 µM shikonin (which had no effect on PARP1 cleavage), almost completely prevented gap closure (82.2 ± 1.3% gap area compared with 38.8 ± 3.8% remaining with mitomycin alone; Fig. 2cii). Collectively these data suggest that PKM2 with shikonin inhibits Mia PaCa-2 cell migration independent of cell growth or cell death.

### Shikonin inhibits glycolysis and causes ATP depletion

**Fig. 3:**
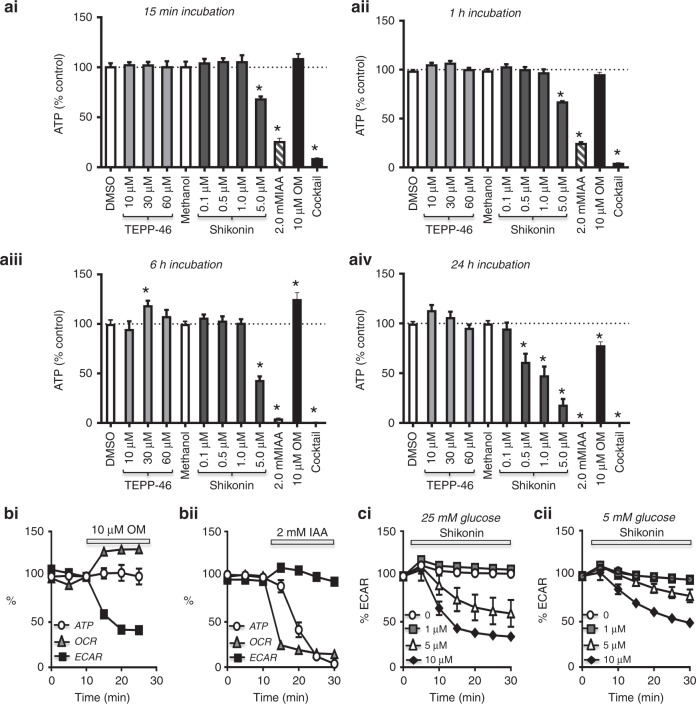
Shikonin induces ATP depletion due to inhibition of glycolysis in PDAC cells. **a** ATP was measured using a 96-well plate based on firefly luciferase luminescence assay in Mia PaCa-2 cells. Cells were treated with different concentrations of PKM2 activator, TEPP-46 (10–60 µM, grey bars), PKM2 inhibitor and shikonin (0.1–10 µM, black bars). DMSO and methanol were used as vehicle controls (white bars), and 2 mM iodoacetate (IAA, hash bars) and 10 µM oligomycin (OM, black bars) were used as positive and negative controls, respectively. ATP luminescence was measured at 15 min (**ai**), 1 h (**aii**), 6 h (**aiii**) and 24 h (**aiv**) post drug treatment. Raw luminescence counts were normalised to an identical corresponding plate stained with SRB prior to normalising to vehicle controls. Significance was determined using a Kruskal–Wallis test with Dunn’s test (*p < 0.05). Data were averaged across multiple replicates (four per experiment) for four experiments. **b** Simultaneous measurement of oxygen consumption rate (OCR, mitochondrial metabolism) and extracellular acidification rate (ECAR, glycolysis), using the Seahorse XF Analyzer, and the quasi-simultaneous, time-matched measurement of cytosolic ATP using GO-ATeam FRET microscopy. Data were normalised to the third control measurement (10 min) prior to addition of 10 µM oligomycin (OM) to inhibit mitochondrial F_1_F_0_-ATP synthase (**bi**) or 2 mM iodoacetate (IAA), to inhibit the glycolytic enzyme GAPDH (**bii**). **c** Concentration-dependent effect of shikonin (1–10 µM) on normalised ECAR (% of control) in Mia PaCa-2 cells cultured in high- (**c**i, 25 mM) or low- (**cii**, 5 mM) glucose DMEM.

Since shikonin inhibited both PDAC cell proliferation and migration, we next aimed to determine whether this was due to specific inhibition of PKM2 and thus depletion of glycolytically derived ATP. We first used a luciferase-based ATP assay to compare the effects of shikonin and TEPP-46 with other metabolic inhibitors.^11^ Shikonin caused a concentration- and time-dependent ATP depletion, but was less effective and slower at depleting ATP compared with the classical glycolytic inhibitor, iodoacetic acid (IAA, Fig. 3ai–v). Only the highest concentration of shikonin (5 µM) induced a significant ATP depletion between 15 min and 6 h (Fig. 3ai–aiii), whereas the lower concentrations (0.5 and 1 µM) induced a significant ATP depletion after 24 h (Fig. 3aiv). In contrast, TEPP-46 had no significant effect on ATP over 24-h treatment. Consistent with previous studies,^10,11^ OM also had no effect on ATP depletion, except after 24 h where a small but significant ATP depletion was observed (Fig. 3aiv).

We next tested whether the shikonin-induced ATP depletion was due to inhibition of PKM2 and thus glycolysis. This was achieved using a Seahorse XFe96 Analyzer, which simultaneously measures oxygen consumption rate (OCR) and extracellular acidification rate (ECAR) as a measure of mitochondrial metabolism and glycolysis, respectively. Initial experiments assessed the effects of the mitochondrial inhibitor OM and glycolytic inhibitor IAA on OCR and ECAR. Over an identical time course, we assessed cytosolic [ATP] in Mia PaCa-2 cells stably expressing the recombinant ATP reporter, GO-ATeam.^11^ This enabled the comparison of the rate of ATP depletion with the rate of change in glycolysis (ECAR) vs mitochondrial metabolism (OCR) (Fig. 3bi and 3bii). All three parameters were normalised to baseline. As expected, OM caused a rapid decrease in OCR accompanied by a compensatory increase in ECAR, which was sufficient to maintain cytosolic [ATP] that remained unaltered (Fig. 3bi). On the other hand, IAA induced a rapid and substantial decrease in ECAR (to 14 ± 1% of baseline, Fig. 3bii), yet any compensatory increase in OCR was minimal and therefore [ATP] rapidly declined (to 4 ± 2% of baseline, Fig. 3bii). This suggests that when glycolysis is inhibited, Mia PaCa-2 cells are unable to upregulate mitochondrial ATP production sufficiently to prevent ATP depletion. We next aimed to test the effects of shikonin on metabolism. However, the spectral properties of shikonin precluded the use of GO-ATeam and measurements of OCR, and thus only ECAR could be accurately assessed. Shikonin caused a concentration- and time-dependent inhibition of ECAR, which was more effective in highly glycolytic cells cultured in high-glucose (25 mM, Fig. 3ci) vs glucose-restricted cells (5 mM, Fig. 3cii). Collectively, these data suggest that shikonin exerts its cytotoxicity by inhibiting glycolysis and inducing ATP depletion, most likely due to inhibition of PKM2.

### PKM2 and other glycolytic enzymes associate with plasma membrane proteins

**Fig. 4:**
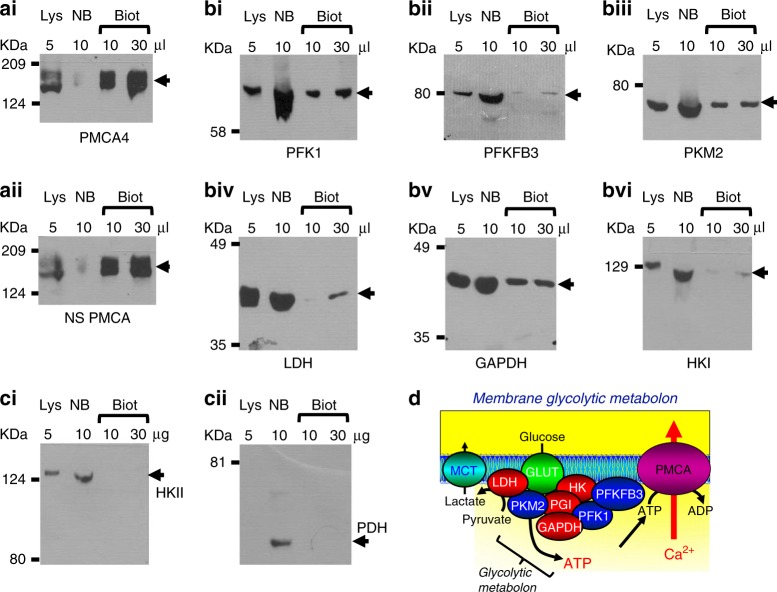
Glycolytic enzymes are associated with plasma membrane proteins in Mia PaCa-2 cells. Mia PaCa-2 cells were incubated with sulfo-NHS-SS-Biotin, which binds to primary amines on cell surface/transmembrane proteins. These biotinylated proteins were separated from non-biotinylated proteins using NeutrAvidin^TM^ agarose beads and eluted using dithiothreitol. Whole-cell lysates (Lys), non-biotinylated (NB) and biotinylated (Biot) fractions were separated by SDS-PAGE and western blotted for the membrane proteins, PMCA4 (**a**i) and non-specific (NS) PMCA (**aii**), the glycolytic enzymes, phosphofructokinase-1 (PFK1, **bi**), phosphofructokinase fructose bisphosphatase-3 (PFKFB3, **bii**) and pyruvate kinase-M2 (PKM2, **biii**), lactate dehydrogenase (LDH, **biv**), glyceraldehyde phosphate dehydrogenase (GAPDH, **bv**), hexokinase-I (HKI, **bvi**) and the mitochondria-associated proteins hexokinase-II (HKII, **ci**) and pyruvate dehydrogenase (PDH, **cii**). **d** Cartoon depicting a complex of key glycolytic enzymes associated with the plasma membrane (membrane glycolytic metabolon) in close proximity to glucose transporters (GLUT), lactate transporters (MCT) and the PMCA.

This was achieved using a cell surface biotinylation assay. We first confirmed that this biotinylated fraction was enriched with bona fide plasma membrane proteins by western blotting for PMCA. As expected, bands for PMCA (~140 kDa), using antibodies for PMCA4 (Fig. 4ai) and non-specific PMCA (NS) (Fig. 4aii), were detected in the biotinylated fraction and whole-cell lysate, but not in the non-biotinylated fraction. Bands were also detected in the biotinylated fraction, non-biotinylated fraction and whole-cell lysate, for key glycolytic enzymes that included platelet phosphofructokinase-1, (PFKP, 80 kDa, Fig. 4bi), 6-phosphofructo-2-kinase/fructose-2,6-biphosphatase 3 (PFKFB3, 60 kDa, Fig. 4bii), pyruvate kinase muscle 2 (PKM2, 60 kDa, Fig. 4biii), lactate dehydrogenase A (LDHA, 37 kDa, Fig. 4biv), glyceraldehyde-3-phosphate dehydrogenase (GAPDH, 37 kDa, Fig. 4bv) and hexokinase-I (HKI, 102 kDa, Fig. 4bvi). However, hexokinase-II (HKII, 102 kDa, Fig. 4ci) and pyruvate dehydrogenase (PDH, 43 kDa, Fig. 4cii) were only detected in the non-biotinylated fraction and/or whole-cell lysate, as they are known to associate with the mitochondria (Fig. 4d). These results suggest that glycolytic enzymes detected in the biotinylated fraction represent a pool of plasma membrane-associated glycolytic enzymes, as illustrated in the cartoon depicting the membrane glycolytic metabolon (Fig. 4d).

### Shikonin induces cytotoxic Ca^2+^ overload and inhibition of PMCA activity in Mia PaCa-2 cells

**Fig. 5:**
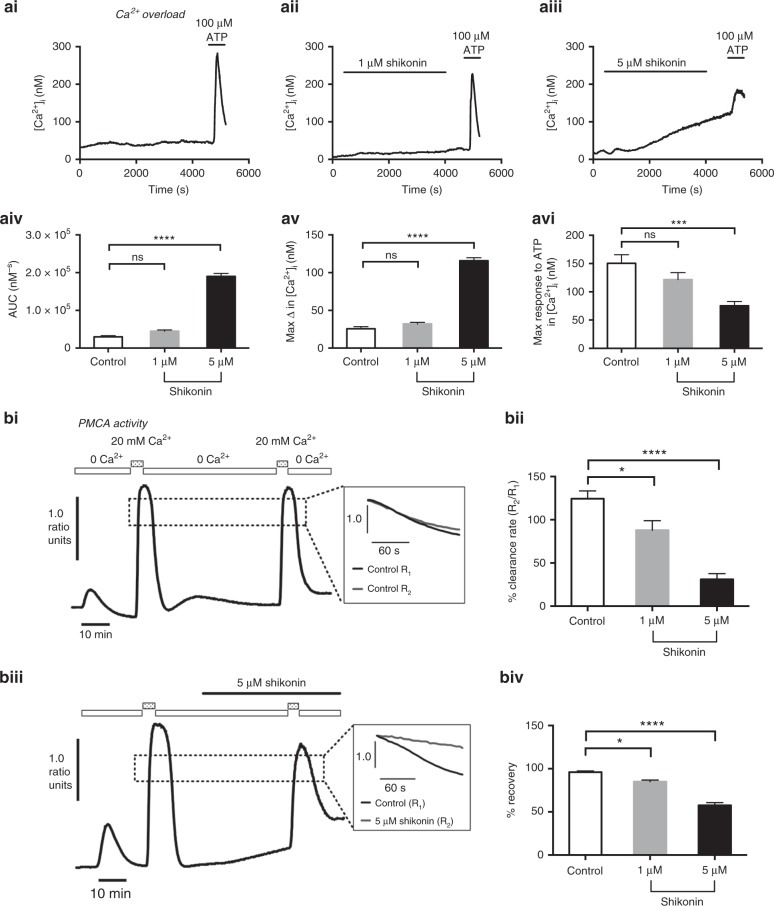
PKM2 inhibitor, shikonin induces a cytotoxic [Ca^2+^]_i_ overload and inhibition of the PMCA in PDAC cells. **a** Cytosolic Ca^2+^ ([Ca^2+^]_i_) was measured in fura-2-loaded Mia PaCa-2 cells and perfused with HEPES-PSS containing shikonin for 1 h, followed by 100 μM ATP to test for cell responsiveness and thus viability. Representative traces showing changes in [Ca^2+^]_i_ following treatment without (time-matched control, **ai**) or with 1 μM shikonin (**aii**) and 5 μM shikonin (**aiii**). Changes in [Ca^2+^]_i_ were quantified and compared by calculating area under curve (AUC) during 1 h of treatment (**aiv**) and maximum change in [Ca^2+^]_i_ before ATP treatment (**av**). Responses of cells to post-treatment ATP were quantified by assessing the maximum change in [Ca^2+^]_i_ after ATP was applied (**avi**). ***p < 0.001; ****p < 0.0001 (one-way ANOVA with Dunnett’s multiple comparisons test). Data were averaged across multiple repeats (13–21 cells per experiment) for five experiments. **b** Effect of shikonin on PMCA activity of Mia PaCa-2 cells. Representative traces show the in situ Ca^2+^ clearance assay of PMCA activity for time-matched control (**bi**) and 5 μM shikonin (**bii**). Cells were treated with 30 μM CPA in zero Ca^2+^ containing 1 mM EGTA (white box) and 20 mM Ca^2+^ (dotted box) to induce Ca^2+^ influx. The influx-clearance phase was repeated giving a paired experimental design, and shikonin was applied during the second influx-clearance phase as shown by the black line in **biii**. Normalised linear clearance rate of the first (R_1_) and the second clearance phase (R_2_) over 60 s from the same starting value was compared (R_2_/R_1_ × 100%) and average data shown in **bii**. **bi** Recovery rate after the second clearance phase was compared with the baseline [Ca^2+^]_i_ between the initial and the second clearance phase and quantified as % recovery (**biv**). Statistical significance was determined using a Kruskal–Wallis test with Tukey’s multiple comparisons, *p < 0.05.

We next aimed to determine whether the shikonin-induced inhibition of glycolysis and consequent ATP depletion led to inhibition of the PMCA and the consequent cytotoxic Ca^2+^ overload. This was achieved by measuring [Ca^2+^]_i_ by fura-2 fluorescence imaging. The higher concentration of shikonin (5 μM) induced a slow, irreversible increase in [Ca^2+^]_i_ compared with time-matched control cells, whereas 1 μM shikonin had no significant effect (Fig. 5a). Moreover, 5 μM shikonin significantly blunted the ATP-induced [Ca^2+^]_i_ response compared with time-matched controls, whereas 1 μM shikonin had no effect (Fig. 5avi). These data indicate that 5 µM shikonin induces an irreversible cytotoxic Ca^2+^ overload within an hour, rendering cells unable to respond to subsequent stimulation with agonists. Shikonin also significantly inhibited PMCA-mediated [Ca^2+^]_i_ clearance to 85 ± 2% (1 µM, Fig. 5bii; *n* = 5) and 58 ± 3% (5 µM, Fig. 5bii and 5biii; *n* = 6), compared with time-matched control cells (124 ± 9%, Fig. 5bi and 5bii; *n* = 4). Moreover, in most cells treated with shikonin, [Ca^2+^]_i_ failed to fully recover (% recovery, Fig. 5biv). Together, these results indicate that shikonin inhibits PMCA activity, which results in the cytotoxic Ca^2+^ overload.

### Effect of PKM2 knockdown on cell proliferation/viability and cell death

**Fig. 6:**
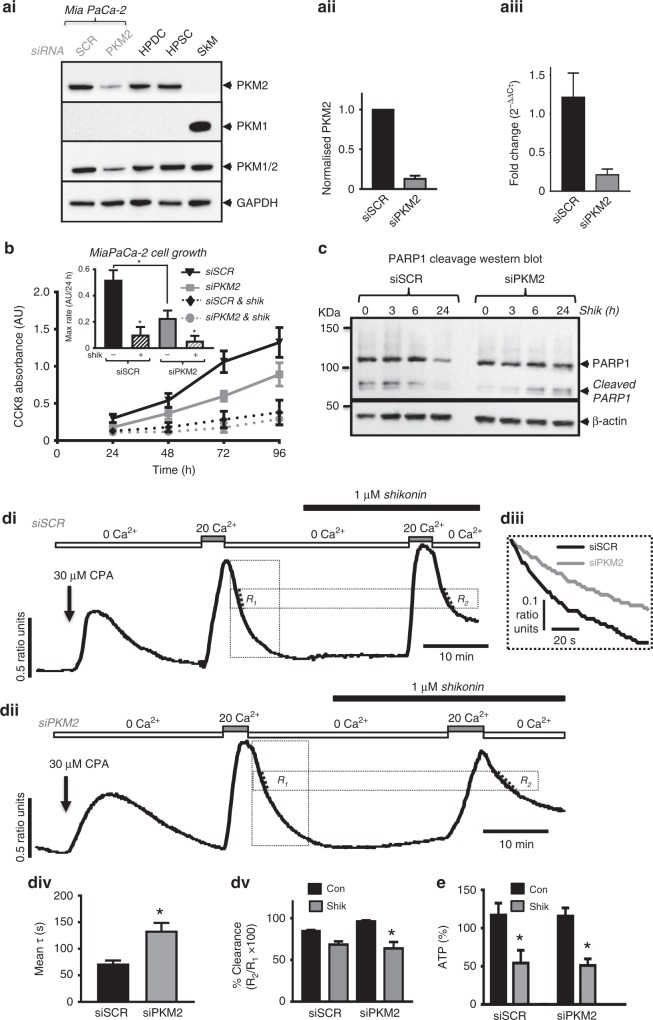
PKM2 siRNA-mediated knockdown reduces cell growth/viability, protects against shikonin-induced PDAC cell death and inhibits PMCA activity. **a** Treatment of Mia PaCa-2 cells with PKM2 siRNA reduces the expression of PKM2 as assessed using western blotting with PKM2-specific antibody (**ai**, **aii**) and qPCR (a**iii**). As additional controls, the same samples were western blotted with a PKM1-specific antibody and a pan-PKM1/2 antibody and compared with lysates from non-cancerous human pancreatic ductal epithelial cells (HPDE) and human pancreatic stellate cells (HPSC). Mouse skeletal muscle (SkM) lysates were used as a positive control for PKM1 expression (**ai**). GAPDH was used as a loading control. **aii** PKM2 protein expression from PKM2 siRNA-treated cells was quantified by densitometry by normalising PKM2 band intensity to the GAPDH loading control before normalising to the band intensity of the scrambled siRNA-treated cells (siRNA) in each gel (*n* = 3 separate experiments). **b** Effect of PKM2 siRNA vs scrambled siRNA with and without shikonin on cell growth/viability using CCK-8 assay (**b** and inset figure) over 96 h. Maximum rate of growth (48–72 h) was compared for each treatment (inset figure). **c** Western blot showing shikonin-induced PARP1 cleavage following 3–24-h treatment with 5 µM shikonin. β-actin was used as a loading control. **d** Representative in situ Ca^2+^ clearance assays to assess PMCA activity in fura-2-loaded MIA PaCa-2 cells treated with scrambled siRNA (siSCR) (**di**) or PKM2 siRNA (siPKM2) (**dii**) for 72 h. Cells were first perfused with HEPES-PSS containing 0 Ca^2+^/1 mM EGTA and 30 μM CPA (arrow) before adding 20 mM Ca^2+^ to induce Ca^2+^ entry. Subsequent removal of external Ca^2+^ (0 Ca^2+^/1 mM EGTA) led to rapid Ca^2+^ clearance and the falling phase of this initial Ca^2+^ clearance phase was fitted to a single exponential to yield a time constant (τ) as a measure of clearance rate. **diii** Expanded time course superimposed for siSCR (black) and siPKM2 (grey). **div** Mean time constant for siSCR (black box) and siPKM2 (grey box). An unpaired *t*-test was used to determine statistical significance. *n* = 7–9 (8–18 cells analysed per experiment) *p ≤ 0.01. Using a paired experimental design, the effect of shikonin during the second Ca^2+^ clearance phase (R_2_) was compared with the initial Ca^2+^ clearance phase (R_1_) for both scrambled siRNA (siRNA) and PKM2 siRNA-treated cells (siPKM2). **div** Mean normalised linear Ca^2+^ clearance (% R_2_/R_1_) measured from the same starting value (as indicated by the dashed lines). *p < 0.05, statistical significance was determined using a Kruskal –Wallis test with Tukey’s multiple comparisons. **e** ATP was measured using a 96-well plate based on firefly luciferase luminescence assay in scrambled siRNA vs PKM2 siRNA-treated Mia PaCa-2 cells treated with or without 5 μM shikonin for 6 h. Raw luminescence counts were normalised to protein assessed using a BCA assay prior to normalising to vehicle controls and expressed as a percentage. *p < 0.05, statistical significance was determined using a one-way ANOVA with Tukey’s multiple comparisons test prior to normalisation.

To examine whether the effects of shikonin were specific to PKM2 inhibition, we knocked down PKM2 using siRNA and assessed the effects on cell proliferation (CCK-8 and SRB assays) and cell death (PARP1 cleavage). Knockdown of PKM2 was first confirmed, and PKM2 protein semi-quantified by western blotting (Fig. 6a, b) and mRNA quantified using qPCR (Fig. 6c). On average, PKM2 protein was reduced to 13 ± 4 (*n* = 3) and mRNA was reduced to 21 ± 7 (*n* = 6). Moreover, there was no detectable expression of PKM1 in scrambled siRNA or PKM2 siRNA-treated cells, compared with mouse skeletal muscle (SkM, Fig. 6a), suggesting that there was no compensatory increase in PKM1 following knockdown of PKM2. PKM2 knockdown significantly reduced the maximum growth rate, as assessed with the CCK-8 assay (Fig. 6b). However, there was no effect using the SRB assay, although there was high variability due to the transfection reagent affecting cell adherence (Fig. S3A,  B). Nevertheless, shikonin further inhibited cell growth using both assays, suggesting that any residual PKM2 expression is further inhibited by shikonin or that shikonin exhibits PKM2-independent effects (Fig. 6b, S3).

To test whether shikonin-induced cell death (Fig. 2bi) was PKM2-dependent, we compared shikonin-induced PARP1 cleavage between scrambled siRNA (siSCR) vs PKM2 siRNA (siPKM2)-treated cells (Fig. 6c). Shikonin (5 μM) induced PARP1 cleavage in scrambled siRNA-treated cells in as little as 3 h, which then declined at 6 and 24 h, possibly due to PARP1 degradation (Fig. 6c). However, in PKM2 siRNA-treated cells, shikonin-induced PARP1 cleavage was reduced and delayed (Fig. 6c). This suggests that the cell death induced by shikonin is PKM2-dependent in the short term, whereas longer-term treatment with shikonin may have PKM2-independent effects.

### PKM2 knockdown inhibits PMCA activity

To test whether PKM2 specifically fuels the PMCA, we compared the PMCA-mediated initial clearance (R_1_) between scrambled (Fig. 6di) vs PKM2 siRNA-treated cells (Fig. 6dii). PMCA-mediated Ca^2+^ clearance was quantified by fitting the clearance curve to an exponential decay, which determined the time constant (τ) that was compared between siSCR vs siPKM2 cells (Fig. 6diii, div). Clearance rate was much slower in siPKM2 cells (i.e. time constant was significantly higher; τ = 145 ± 9 s, *n* = 4; Fig. 6diii, div) vs siSCR cells (τ = 103 ± 4 s, *n* = 4; Fig. 6diii, div). Collectively, these data suggest that PKM2 is important for maintaining PMCA activity and when PKM2 expression is reduced, PMCA activity is reduced.

However, it is important to note that PMCA was still able to clear cytosolic Ca^2+^ back to baseline, albeit at a much slower rate, suggesting that the residual PKM2 expression following siRNA-mediated knockdown may be sufficient to maintain PMCA activity to some extent. However, using a paired experimental design in which shikonin was applied during the second [Ca^2+^]_i_ clearance phase, shikonin was able to further inhibit PMCA activity to a similar extent in PKM2 siRNA-treated cells vs scrambled siRNA-treated cells (Fig. 6d). This may seem counterintuitive, as one might expect PKM2 knockdown to prevent shikonin-induced inhibition of the PMCA. However, it is important to note that these paired experiments compare the effect of shikonin on relative [Ca^2+^]_i_ clearance compared with the initial clearance that was already slowed by knockdown of PKM2. However, it was not possible to fit this shikonin-induced reduced [Ca^2+^]_i_ clearance to a single exponential decay as [Ca^2+^]_i_ almost always recovered to a new elevated baseline (asymptote). Nevertheless, these data reinforce the notion that shikonin further inhibited the remaining residual PKM2 following siRNA-mediated knockdown. Consistent with PMCA-mediated [Ca^2+^]_i_ clearance, similar results were obtained with ATP depletion (Fig. 6e); shikonin treatment (5 μM for 6 h) induced a similar ATP depletion in PKM2 siRNA-treated cells (51 ± 9, n = 4) vs scrambled siRNA-treated cells (54 ± 17, *n* = 4; Fig. 6e). This also suggests that the residual PKM2 expression is sufficient to maintain cellular ATP and thus PMCA activity, and that inhibition of this residual PKM2 by shikonin was able to induce further ATP depletion.

## Discussion

The current study demonstrates that PKM2 overexpression in human PDAC is associated with poor survival. Moreover, PKM2 with shikonin inhibited numerous cancer hallmarks in pancreatic cancer cells, including cell growth/proliferation, cell viability, cell migration and induction of cell death. This was due in part to inhibition of glycolysis, ATP depletion, inhibition of PMCA activity and cytotoxic Ca^2+^ overload. Moreover, knockdown of PKM2 expression using siRNA reduces cell growth/viability, reduced shikonin-induced cell death and reduced PMCA activity. However, despite PKM2 knockdown reducing total PKM2 protein expression to as low as 13%, this residual PKM2 was sufficient to maintain cellular ATP and thus PMCA, albeit at a reduced activity, whereas further treatment with shikonin was capable of further inhibiting this residual PKM2, thereby inducing ATP depletion and further inhibition of the PMCA.

Furthermore, cell surface biotinylation assays identified that numerous glycolytic enzymes (including PKM2) associate with plasma membrane protein(s), presumably in close proximity to the PMCA. Collectively, these data suggest that PKM2 is critical for numerous cancer hallmarks, consistent with previous studies,^12,14,15,30–33^ and that a pool of PKM2 may form part of a metabolic hub (glycolytic metabolon) at the inner envelope of the plasma membrane that provides a privileged ATP supply to the PMCA. Such a mechanism may represent a novel therapeutic target; disruption of this sub-membrane glycolytic metabolon could cut off the privileged ATP supply to the PMCA, thereby inducing cytotoxic Ca^2+^ overload and cell death.

PKM2 is regarded as one of the major oncogenic glycolytic enzymes and is abundantly expressed in numerous highly proliferative cells such as cancer cells.^12,14,15,30–33^ Although phosphoglycerate kinase is another ATP-generating enzyme, this enzyme is the first step of the “pay off” phase of glycolysis, which follows the preparatory steps of glycolysis (hexokinase (HK) and phosphofructokinase-1 (PFK1)), which consume ATP and therefore does not produce any net ATP. In other words, the ATP produced by phosphoglycerate kinase balances the ATP that is consumed by HK and PFK1. This means that there is no net ATP produced until the PKM2 step and therefore PKM2 is the major ***net*** ATP-generating glycolytic enzyme in PDAC cells and thus critical for fuelling the PMCA that is relevant to the current study. Moreover, PKM2 predominantly exists in its dimeric form in cancer cells, whereas in non-cancer cells, it exists as a tetramer, with similar functional properties to PKM1.^34^ Dimeric PKM2 has a lower catalytic activity, which results in a bottleneck at the terminal end of glycolysis and thus a buildup of biosynthetic glycolytic intermediates upstream of PKM2, which are required for rapidly dividing cancer cells. Moreover, dimeric PKM2 is maintained by tyrosine phosphorylation,^34^ and other post-translational modifications,^35–38^ all of which tend to be upregulated in cancer cells due to overexpression of growth factor receptors and mutant KRas. However, this reduced catalytic activity of PKM2 results in reduced ATP production, which combined with impaired mitochondrial function, makes cancer cells bioenergetically compromised compared with normal non-cancerous cells. It therefore makes good teleological sense for PKM2 to localise to where ATP is required, such as at the plasma membrane in close proximity to the PMCA. Indeed, our cell surface biotinylation assays showed that numerous glycolytic enzymes associated with the plasma membrane. Previous studies in erythrocytes, which lack mitochondria, show a similar plasma membrane-localised complex of glycolytic enzymes that bind to anion exchanger-1 (AE1).^39,40^ This sub-membrane pool of glycolytic enzymes filled a cytoskeletal compartment with ATP that preferentially fuelled the PMCA without direct binding.^19^ More recently, a membrane-bound pool of PKM2 has been reported to be important for regulating cell–cell junctions and migration in endothelial cells, presumably by providing a privileged ATP supply similar to the present study.^41^

So what is the functional significance of plasma membrane-associated glycolytic enzymes? Firstly, this would improve the efficiency of glucose metabolism and lactic acid efflux, not only due to the proximity of glucose transporters and lactic acid transporters at the membrane, but also due to “substrate channelling”.^42,43^ Secondly, the presence of the glycolytic machinery at the plasma membrane provides a privileged ATP supply to energy-consuming processes at the plasma membrane, which include the Na^+^/K^+^ ATPase,^19,44,45^ cell migratory machinery^41,46^ as well as the PMCA.^20,47,48^ More recent studies have shown that activation of the Na^+^/K^+^ ATPase stimulates a corresponding increase in glycolytic rate, whereas its inhibition with ouabain results in a decrease in glycolytic rate, supporting the notion that it is glycolysis that supports membrane pumps. Finally, ion pumps are major ATP consumers, utilising between 20 and 50% of total ATP consumption.^49^ Moreover, the rate-limiting glycolytic enzyme PFK1 is inhibited by high [ATP]^50^ and high [Ca^2+^].^51^ Therefore, co-localisation of glycolytic enzymes with the PMCA, not only provides a privileged ATP supply to the PMCA, but also maintains [ATP] and [Ca^2+^] below the inhibitory threshold of PFK1, thereby maintaining glycolytic flux and a Warburg phenotype.

The present study also found that shikonin reduced PDAC cell growth, migration and induced cell death, presumably due to inhibition of PKM2 because it also caused a dramatic inhibition of glycolysis and ATP depletion. Moreover, shikonin also inhibited the PMCA and induced cytotoxic Ca^2+^ overload, providing a potential functional link between PKM2 and PMCA activity. Shikonin is a naturally occurring naphthoquinone derivative that is extracted from Zicao roots.^52^ It has been used as a traditional Chinese herbal medicine for its antimicrobial, anti-inflammatory and numerous anticancer effects.^21–25^ Moreover, shikonin exhibits a 10–20-fold selectively for PKM2 compared with PKM1 and PKL.^21^ Specifically, using in vitro assays of pyruvate kinase (PK) activity, the IC_50_ for PKM2 was found to be ~0.5 μM compared with 10 μM for PKM1 and 5 μM for PKL. In addition, shikonin inhibited glucose consumption and lactate production with an IC_50_ of 5–10 μM in MCF-7 cells that exclusively express PKM2, and thus acts as an indirect surrogate functional assay of PKM2 activity. Therefore, by extrapolation of these in vitro and functional assays, it is unlikely that the effects of shikonin, used at a concentration in the present study (0.1–5 μM), are due to inhibition of other pyruvate kinase isoforms. However, there are numerous additional reported effects of shikonin such as regulation of growth factor signalling, phosphorylation of apoptosis-inducing proteins^24^ and inhibition of Akt and RIP1/NFkB,^22^ which activate p53 and p24.^53^ While some of these effects may be a downstream consequence of PKM2 inhibition, some may be PKM2-independent. Nevertheless, it is also worth noting that most of these effects occur at higher concentration of shikonin (supramicromolar) and longer treatment periods (>24 h), where there may be changes in gene expression.^26^ Therefore, these could not explain the acute effects of shikonin treatment on inhibition of glycolysis, ATP depletion, inhibition of the PMCA and cytotoxic Ca^2+^ overload in the present study. Shikonin is also reported to impair mitochondrial function and increase oxidative stress,^52,54^ although in the context of bioenergetics, our previous studies show that inhibition of mitochondria has minimal effects on ATP depletion, cytotoxic Ca^2+^ overload or inhibition of the PMCA.^10,11^ Therefore, despite some of the reported non-specific effects of shikonin, on balance we would strongly argue that all effects of shikonin reported in the present study, particularly inhibition of PMCA activity, are most likely to be due to inhibition of PKM2. Shikonin is also relatively free from cancer drug resistance,^53^ suggesting that it may be translated clinically to treat PDAC patients.

Although we cannot completely rule out additional PKM2-independent effects of shikonin in the present study, the functional effects of PKM2 siRNA, on the other hand, can be directly ascribed to the specific knockdown of PKM2 expression. PKM2 siRNA reduced cell growth (as assessed using the tetrazolium-based CCK-8 assay), reduced PMCA activity and protected against shikonin-induced cell death. The reduced PMCA-mediated Ca^2+^ clearance following treatment with PKM2 siRNA in the absence of shikonin provides the most compelling and direct evidence that PKM2 provides a privileged ATP supply to the PMCA and eliminates any confounding potential non-specific effects of shikonin.

This therefore further supports the general notion that PKM2, or more specifically the PKM2–PMCA functional coupling, might represent a novel therapeutic target. The design of novel small molecules/peptides that disrupt this functional coupling, for example by disrupting the binding of PKM2 to the putative plasma membrane protein(s), would be predicted to cut off the privileged ATP supply to the PMCA and thus induce cytotoxic Ca^2+^ overload and cell death.

Although PKM2 is reported to be highly oncogenic in numerous cancers,^14,15,30–33^ there are several strands of evidence from cell lines and genetically modified mouse models of different cancers that PKM2 deletion has no effect on cancer.^34,37,55–59^ More specifically, a recent study suggests that PKM2 is redundant in a mouse model of PDAC (LSL-KrasG12D/+;Trp53flox/flox;Pdx-1-Cre (KP−/−C) mice).^60^ This seems counterintuitive to the large body of evidence supporting an oncogenic role of PKM2 described above. However, it has been recently argued that the low pyruvate kinase activity is what drives tumorigenicity^61^ and this in turn is dependent on the relative amount of dimeric PKM2 vs tetrameric PKM,^34,62^ rather than the absolute expression of total PKM2 protein. Indeed, this was evidenced by siRNA-mediated PKM2 knockdown in the present study, which did not always produce clear-cut results and presented an important functional paradox. This is because the residual PKM2 expressed after siRNA-mediated PKM2 knockdown appears sufficient to maintain ATP and PMCA activity, albeit at a reduced rate, and subsequent treatment with shikonin inhibited this residual PKM2, causing a similar ATP depletion and further inhibition of PMCA. This seems counterintuitive, as one would predict that PKM2 knockdown should reduce the effects of shikonin on ATP production and PMCA activity, if indeed these effects are specific for PKM2. However, this can be reconciled by the fact that total PKM2 protein is less important than total pyruvate kinase activity for ATP production and PMCA activity, which in turn is dependent on the relative dimeric vs tetrameric PKM2. Therefore, despite a significant reduction in total PKM2 protein, there may be a shift from predominantly dimeric PKM2 with low pyruvate kinase activity to tetrameric PKM2 with high PK activity, which may be sufficient to preserve cellular ATP production and thus PMCA activity to some extent.

Moreover, dimeric PKM2 can be maintained by tyrosine phosphorylation^34^ and other post-translational modifications.^35–38^ Cancer cells are also particularly adept at re-wiring their metabolism following gene deletion, and indeed PKM1 activity may compensate for the loss of PKM2 in many of the above studies.^57,60^ No compensatory increase in PKM1 expression was observed in the present study. Nevertheless, the central tenet of our current study remains valid; the acute drug-induced inhibition of PKM2 rapidly cuts off the ATP supply to the PMCA, inducing cytotoxic Ca^2+^ overload and cell death, and as such any compensatory changes in gene expression (PKM1, or otherwise) would be too slow to compensate.

The idea of localised ATP supply or an ATP microdomain is controversial^63^ because in most cells, global ATP concentration is always well above the saturating concentration for most ATP-dependent processes, including the PMCA.^64,65^ However, such a privileged ATP supply to the PMCA may become critical when ATP synthesis is compromised, for example during severe hypoxia, or in cancer cells that exhibit impaired mitochondrial ATP production or express dimeric PKM2 with low catalytic activity. Therefore, such a strategy of cutting off the glycolytic ATP supply to the PMCA in pancreatic cancer cells would be predicted to be relatively more effective in cancer cells while leaving non-cancerous cells that rely on catalytically active pyruvate kinase (PKL or PKM1) or mitochondria as the major ATP supply, relatively intact.

## Supplementary information

Supplementary Data

## Data Availability

All analysed and derivative raw data are available on request.
