# Supplementary material for: Cutting off the fuel supply to calcium pumps in pancreatic cancer cells: role of pyruvate kinase-M2 (PKM2)
Source: Br J Cancer. 2019 Dec 10;122(2):266–78. doi: 10.1038/s41416-019-0675-3 (PMC7052184; doi:10.1038/s41416-019-0675-3)
Supplement: Supplementary file 1 — Supplementary Data [file 41416_2019_675_MOESM1_ESM.docx]

**Cutting off the fuel supply to calcium pumps in pancreatic cancer cells: Role of pyruvate kinase-M2 (PKM2)**

**Running title:**

**Pyruvate kinase-M2 and pancreatic cancer**

**Andrew D. James^1^, In-Whan Oh, Pishyaporn Sritangos, Daniel A. Richardson, Thomas Attard, Lisa Barrett and Jason I.E. Bruce*.**

Division of Cancer Sciences, Faculty of Biology, Medicine & Health Sciences, The University of Manchester, Michael Smith Building, Manchester, M13 9PT, UK

**^1^**Current address: Division of Cancer Sciences, Department of Biology, University of York, Heslington, York, YO10 5DD, UK.

***Corresponding Author –**

Dr. Jason Bruce

**e-mail:** jason.bruce@manchester.ac.uk

**Tel:** +44 (0)161 275 5484

**Conflicts of Interest –** The authors declare no conflicts of interest.

**Supplementary Data**

**
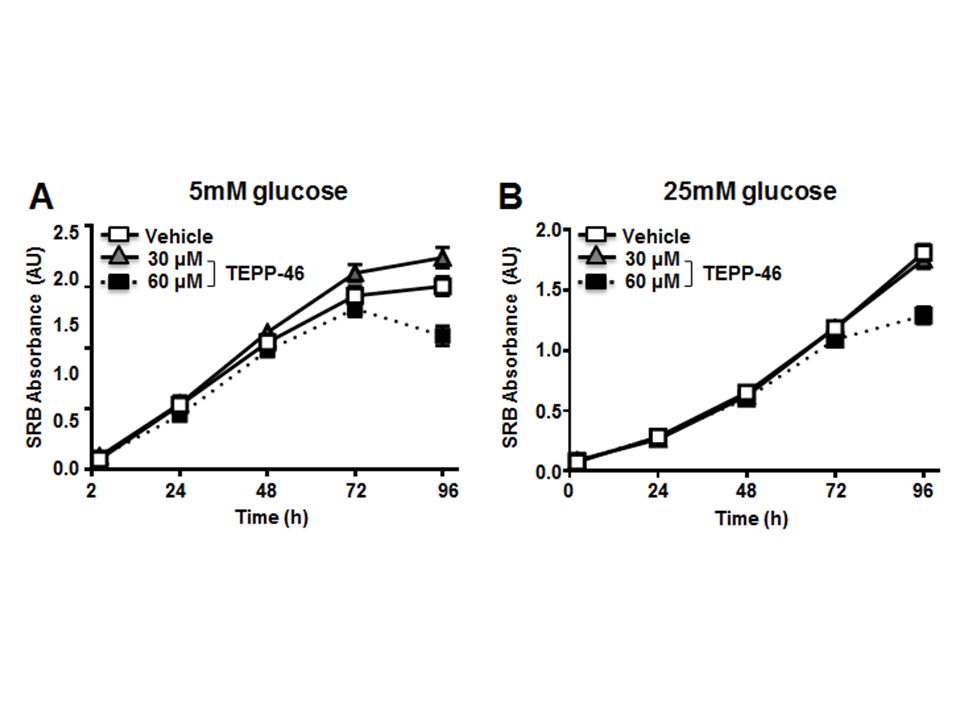
**

**Figure S1.** **Effect of PKM2 activator, TEPP-46, on Mia PaCa-2 cell growth.** Mia-PaCa-2 cells were cultured either in 5mM (***Ai***) or 25mM (***Aii***) glucose containing DMEM and treated with different concentrations of PKM2 activator, TEPP-46 for up to 96 hours. Cell growth was measured using a sulforhodiamine B assay (SRB absorbance unit (AU) at 2 hour and every 24 hours. Data were averaged across multiple repeats (8-16 per experiment) for 3-6 experiments (± SEM).


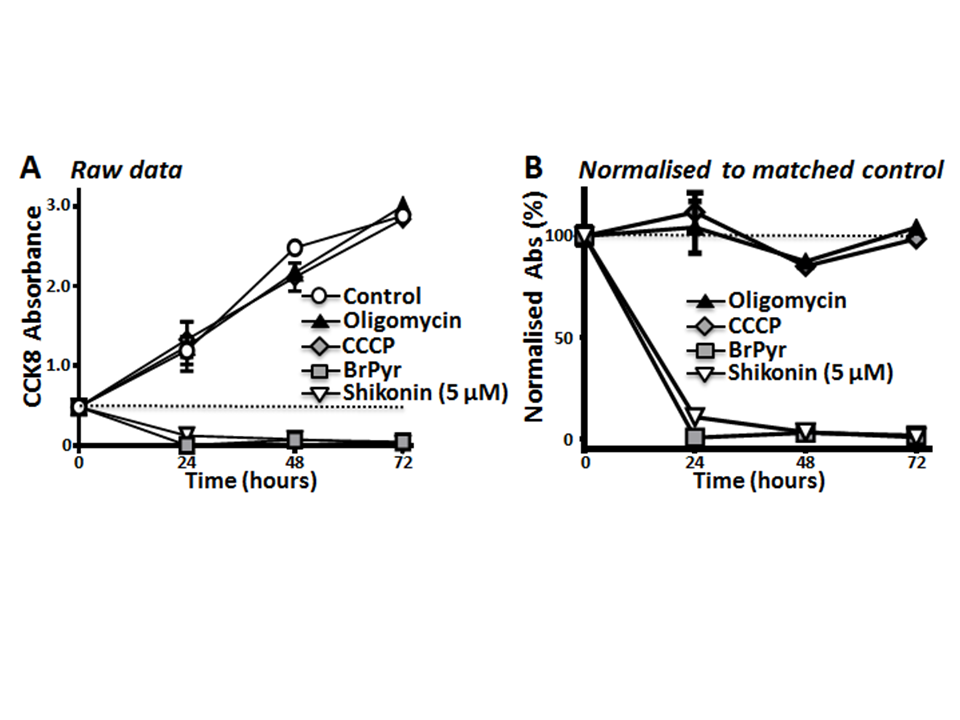


**Figure S2. The PKM2 inhibitor, shikonin, reduces PDAC cell viability. *A,*** Mia PaCa-2 cells were seeded into 96 well plates and a tetrozolium-based substrate (WST) added at 24, 48, 72 and 96 hours to asses cell growth/viability (cell counting kit (CCK8)) following treatment with the mitochondrial inhibitors, oligomycin (10 µM) and CCCP (4 µM), the classical glycolytic inhibitor bromopyruvate (BrPyr; 500 µM) and the PKM2 inhibitor shikonin (5 µM). The CCK-8 assay assesses the redox potential of cells and is thus a measure of cell viability. **A**, The increase in raw absorbance over time (96 hours) reflects cell growth, whereas a decrease in absorbance below the baseline reflects reduced cell viability or cell death. **B**, Raw absorbance normalised to the corresponding time-matched controls (%) show that oligomycin and CCCP have no effect, whereas BrPyr and shikonin reduced cell viability or induced cell death.

**
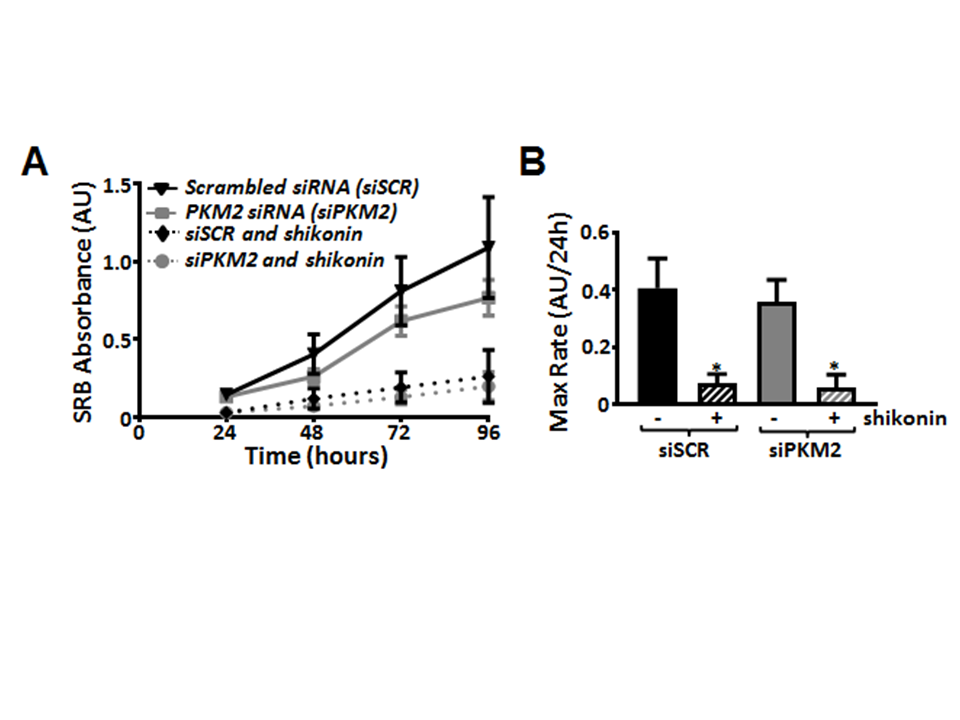
**

**Figure S3. PKM2 siRNA-mediated knockdown reduces cell growth/viability.** ***A.***, Effect of PKM2 siRNA vs scrambled siRNA with and without shikonin on cell growth/viability using CCK-8 assay (***A*** and ***B***) over 96 hours. Maximum rate of growth (48-72 hours) was compared for each treatment (***B***).
